# Factors affecting the clinical response to National Early Warning score triggers

**DOI:** 10.1186/cc13235

**Published:** 2014-03-17

**Authors:** I Kolic, S McCartney, S Crane, Z Perkins, A Taylor

**Affiliations:** 1 South London Healthcare NHS Trust, London, UK; 2 East Kent Hospitals University NHS Trust, Ashford, UK

## Introduction

We aimed to assess actions taken in response to variations in the National Early Warning (NEW) score and to identify factors associated with a poor response. The NEW score is a physiological score, which prescribes an appropriate response for the deteriorating patient in need of urgent medical care. This allows enhanced observation and clinical review of patients, identifying patients at risk of acute mortality.

## Methods

We performed a prospective observational study of adult patients admitted to an acute medical ward in a London district general hospital over a 2-week period. Patient characteristics, NEW score, time of day, day of week and clinical response data were collected for the first 24 hours of admission. Patients with less than a 12-hour hospital stay were excluded. The primary outcome measure was the quality of clinical response. Data were analysed with univariate and multivariate logistic regression.

## Results

During the study period 200 patients were included with a median age of 70 (20 to 102) years. NEW scores were evenly distributed between day and night (52% vs. 48%) with a greater proportion on weekdays compared with weekend days (82% vs. 18%). The majority of patients scored <5 (93% vs. 7%). Forty-seven (27%) patients received an inadequate clinical response. Univariate analysis showed no association with time of day (night 34% vs. day 38%, OR 0.83 (0.47 to 1.49), *P *= 0.556). However, day of the week (weekend 56% vs. weekday 32%, OR 2.8 (1.30 to 5.84), *P *= 0.01) and increasing score (NEWS ≥5 100% vs. NEWS <5 31%, OR 65 (3.8 to 1100), *P *< 0.0001) were significantly associated with an inadequate response. Day of the week was independently associated with an inadequate response after adjusting for confounders (OR 3.08 (1.27 to 7.46), *P *= 0.013).
